# Denitrification, Nitrogen Uptake, and Organic Matter Quality Undergo Different Seasonality in Sandy and Muddy Sediments of a Turbid Estuary

**DOI:** 10.3389/fmicb.2020.612700

**Published:** 2021-01-21

**Authors:** Marco Bartoli, Daniele Nizzoli, Mindaugas Zilius, Mariano Bresciani, Antonio Pusceddu, Silvia Bianchelli, Kristina Sundbäck, Arturas Razinkovas-Baziukas, Pierluigi Viaroli

**Affiliations:** ^1^ Department of Chemistry, Life Sciences and Environmental Sustainability, University of Parma, Parma, Italy; ^2^ Marine Research Institute, University of Klaipeda, Klaipeda, Lithuania; ^3^ Department of Life Sciences and Biotechnology, University of Ferrara, Ferrara, Italy; ^4^ Optical Remote Sensing Group, CNR-IREA, Milan, Italy; ^5^ Department of Life and Environmental Sciences, University of Cagliari, Cagliari, Italy; ^6^ Department of Life and Environmental Sciences, Polytechnic University of Marche, Ancona, Italy; ^7^ Department of Marine Sciences, University of Gothenburg, Gothenburg, Sweden

**Keywords:** Curonian Lagoon, nitrogen, sediment, benthic fluxes, denitrificafion, microphytobenthos, organic matter quality

## Abstract

The interaction between microbial communities and benthic algae as nitrogen (N) regulators in poorly illuminated sediments is scarcely investigated in the literature. The role of sediments as sources or sinks of N was analyzed in spring and summer in sandy and muddy sediments in a turbid freshwater estuary, the Curonian Lagoon, Lithuania. Seasonality in this ecosystem is strongly marked by phytoplankton community succession with diatoms dominating in spring and cyanobacteria dominating in summer. Fluxes of dissolved gas and inorganic N and rates of denitrification of water column nitrate (D_w_) and of nitrate produced by nitrification (D_n_) and sedimentary features, including the macromolecular quality of organic matter (OM), were measured. Shallow/sandy sites had benthic diatoms, while at deep/muddy sites, settled pelagic microalgae were found. The OM in surface sediments was always higher at muddy than at sandy sites, and biochemical analyses revealed that at muddy sites the OM nutritional value changed seasonally. In spring, sandy sediments were net autotrophic and retained N, while muddy sediments were net heterotrophic and displayed higher rates of denitrification, mostly sustained by D_w_. In summer, benthic oxygen demand increased dramatically, whereas denitrification, mostly sustained by D_n_, decreased in muddy and remained unchanged in sandy sediments. The ratio between denitrification and oxygen demand was significantly lower in sandy compared with muddy sediments and in summer compared with spring. Muddy sediments displayed seasonally distinct biochemical composition with a larger fraction of lipids coinciding with cyanobacteria blooms and a seasonal switch from inorganic N sink to source. Sandy sediments had similar composition in both seasons and retained inorganic N also in summer. Nitrogen uptake by microphytobenthos at sandy sites always exceeded the amount loss *via* denitrification, and benthic diatoms appeared to inhibit denitrification, even in the dark and under conditions of elevated N availability. In spring, denitrification attenuated N delivery from the estuary to the coastal area by nearly 35%. In summer, denitrification was comparable (~100%) with the much lower N export from the watershed, but N loss was probably offset by large rates of N-fixation.

## Introduction

Estuaries at the interface between agricultural watersheds and marine coastal ecosystems receive large quantities of anthropogenic nitrogen (N) inputs (Boyer and Howarth, 2008; Grizzetti et al., 2012; Viaroli et al., 2018; Vybernaite-Lubiene et al., 2018). The passive flushing of these inputs offshore can be contrasted by microbial denitrification or slowed *via* uptake and temporary retention of N by primary producers (Crowe et al., 2011; Vybernaite-Lubiene et al., 2017; Tassone and Bukaveckas, 2019). A major fraction of anthropogenic N loads is transported from rivers into estuaries during high-discharge periods, mostly as nitrate (NO_3_^−^; Sigleo and Frick, 2007; Viaroli et al., 2018; Vybernaite-Lubiene et al., 2018) and may simultaneously sustain elevated rates of primary production and denitrification (Raven and Taylor, 2003; Bartoli et al., 2012; Magri et al., 2020).

During low-discharge periods, decreased N export from watersheds enhances the competition between primary producers and bacterial communities in estuaries, resulting in limited N losses (Welsh et al., 2000; Risgaard-Petersen, 2003; Bartoli et al., 2008). However, N-limitation and primary producers-bacteria competition can be offset by elevated recycling from sediments (Wasmund et al., 2001; Fulweiler and Nixon, 2012; Zilius et al., 2018). While these aspects have been analyzed in detail in vegetated lagoons and coastal sediments, they are overlooked in turbid estuarine systems, where benthic primary production is strongly limited by light and considered to be insignificant (Barnes and Owens, 1999; Ottosen et al., 1999; Russell et al., 2016).

In eutrophic estuaries, turbidity is due to resuspension or repeated phytoplankton blooms, often sustained by different algal communities. During the high discharge spring period, large N and silica (Si) transport may favor diatom blooms, whereas cyanobacteria may dominate the algal community during the low discharge summer period. Buoyancy and N-fixation capacity provide indeed a competitive advantage to cyanobacteria under N-limited and turbid conditions (Paerl and Otten, 2013). Settled algae may be able to exploit even small irradiance or remain viable for long periods in the dark, perform dark assimilation, compete with or produce inhibitory substances for microbial communities, and affect N-related microbial processes as denitrification also in turbid systems (Bartoli et al., 2003; Risgaard-Petersen, 2003).

Post-bloom settling of phytoplankton enriches sediments with organic matter (OM). A moderate organic enrichment may stimulate denitrification, whereas a large enrichment may lead to oxygen (O_2_) depletion (Hagy et al., 2004; Diaz and Rosenberg, 2008), suppress the activity of large-sized macrofauna and nitrifying bacteria, and favor NO_3_^−^ ammonification over denitrification (Conley et al., 2009; Gardner and McCarthy, 2009; Magri et al., 2020) with a positive feedback for coastal eutrophication (Fulweiler and Nixon, 2012).

As for many other benthic processes, the food availability of organic substrates for benthic microbial growth and activity is likely another important regulating factor for N transformations in the sediment (Pusceddu et al., 2009). Denitrification, a key process for the estuarine N filter, generally displays better correlation with sediment O_2_ demand rather than with the sedimentary OM load (Seitzinger, 1994). However, such correlation was demonstrated to vary depending on the *δ* ^13^C value and C:N ratio of the OM, suggesting that the origin and the quality OM are important but understudied regulators of denitrification (Eyre et al., 2013). The quality of the OM as regulating factor appears to be crucial particularly in estuaries, where the relative importance of labile (i.e., biopolymers, including proteins, lipids, and low-molecular-weight carbohydrates) over refractory (i.e., humic and fulvic acids, structural carbohydrates, and “black” carbon) compounds can control the transfer of energy through the microbial loop towards higher trophic levels (Pusceddu et al., 2009).

In this study, benthic primary production, the seasonal rates of denitrification and inorganic N fluxes, and the quantity and biochemical composition of OM were studied in the sediments of a turbid, mostly freshwater estuary, the Curonian Lagoon (Lithuania). This lagoon displays pronounced seasonal variations of nutrient loads and stoichiometry (from N and Si excess in spring to N and Si limitation in summer) and phytoplankton community composition (from being diatom-dominated in spring to being cyanobacteria-dominated in summer) and two main sedimentary types (e.g., sandy and muddy; Pilkaitytė and Razinkovas, 2006; Bartoli et al., 2018; Vybernaite-Lubiene et al., 2018; Zilius et al., 2018). Nutrient and light limitations affect the nutritional value of phytoplankton, stimulating lipid synthesis and content in algal cells and in sediments upon settling, with unknown consequences on microbial activities (Harrison et al., 1990; Zeng et al., 2010). The rationale of this study was to contrast two seasons and two sediment types. The first season was early spring after the ice cover melting (March), characterized by low temperatures and presence of large freshwater N input and moderate settling of diatoms. The second season was summer (July) characterized by high temperatures, limited freshwater N loading, and elevated settling of phytodetritus mainly consisting of cyanobacteria. The two sediment types were sand (1-m depth) and mud (2.5-m depth). We hypothesized that benthic N cycling in boreal eutrophic estuaries, like the Curonian Lagoon, undergoes a pronounced seasonal shift. In particular, we expected no competition between benthic microalgae and denitrifying bacteria during spring due to large N excess, and a strong inhibition of both algal uptake and denitrification during summer, due to high turbidity, low N concentrations, and chemically reduced sediments. We also hypothesized a pronounced seasonal shift in the quantity and biochemical composition of sedimentary OM, affecting denitrification and the source/sink function of sediments.

## Materials and Methods

### Study Area

**Figure 1:**
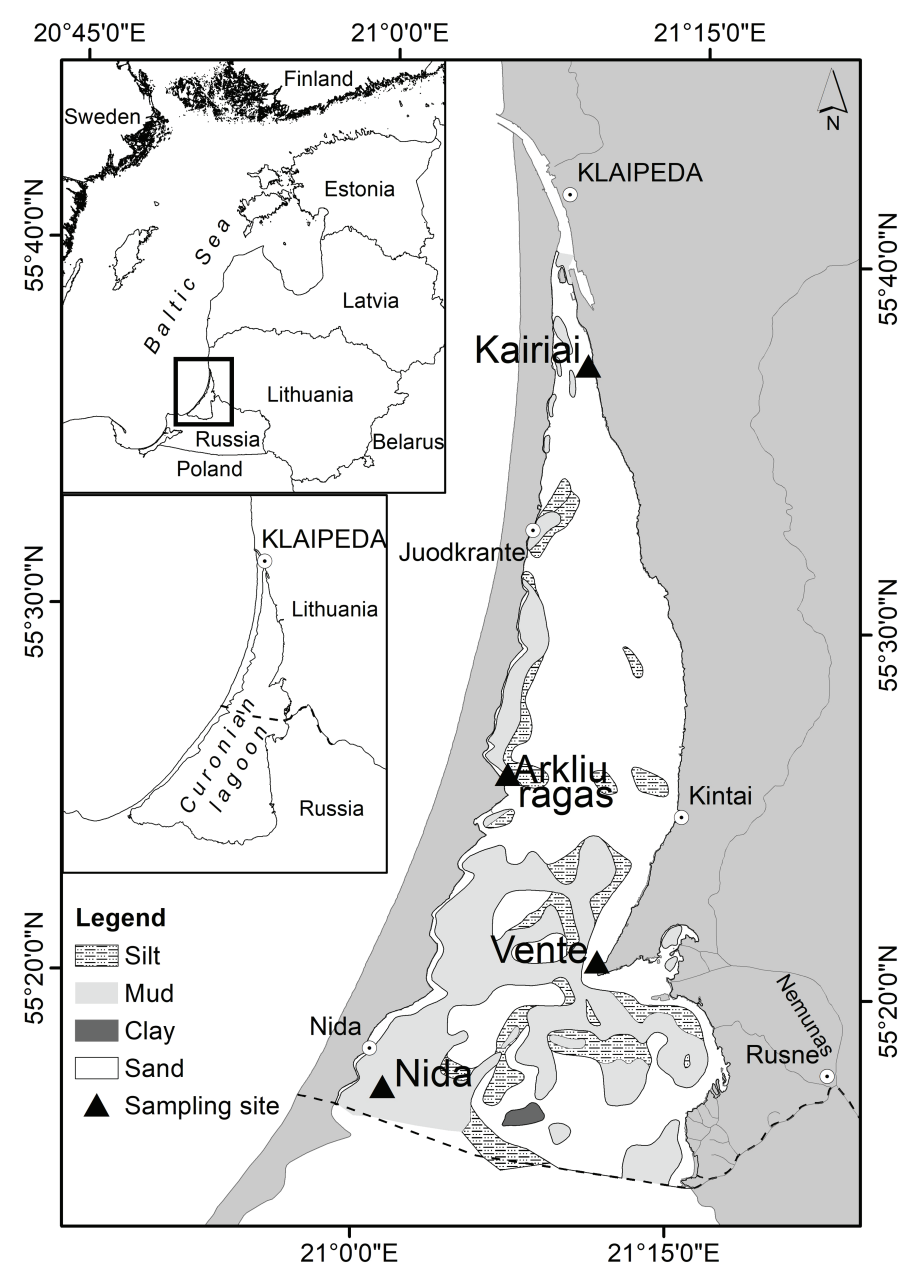
The Curonian Lagoon with the four sampling sites. The dotted line in the central map separates the southern, Russian side of the lagoon from the Lithuanian one.

The Curonian Lagoon is a large (1,584 km^2^), shallow (mean depth 3.8 m), highly eutrophicated and mainly freshwater estuary connected to the Baltic Sea by a narrow strait (Figure 1). Occasionally, wind-driven intrusion of brackish water increases salinity up to 1–2, but only in the northern part of the lagoon. The Nemunas River brings 96% of the total freshwater inputs (annual discharge 21.8 km^3^; Jakimavičius and Kriaučiūnienėe, 2013) and enters the lagoon in its central area. The water body is divided in a north-eastern, transitional, riverine-like part exporting freshwater to the sea and in confined, southern and north-western parts with long water residence time (Ferrarin et al., 2008; Umgiesser et al., 2016). The annual range of water temperatures is wide, from 1°C up to 25–29°C; the strait is permanently ice free, whereas the rest of the lagoon can be ice covered for up to 100 days per year (Žaromskis, 1996).

Shallowness, wind regimes, sediment resuspension, and frequent phytoplankton blooms make this system turbid (Giardino et al., 2010). The nutrients dynamic is similar to that of temperate and boreal transitional waters subjected to seasonal riverine inputs; the highest concentrations are observed in early spring, just after ice melting, and the lowest during summer (Vybernaite-Lubiene et al., 2018). The seasonal succession of the phytoplankton assemblages consists of spring diatom blooms followed by summer blooms of cyanobacteria (Pilkaitytė and Razinkovas, 2006; Zilius et al., 2014). Bottom sediments are a mosaic of sand and silt, with muddy areas on the central and western border, along the Curonian Spit (Trimonis et al., 2003; Bartoli et al., 2020).

### Sampling Strategy

Benthic processes were studied at four sites during two field campaigns carried out between 23 and 29 March and between 20 and 26 July 2009 (Figure 1). Two sites (Arkliu Ragas and Nida) had soft, fine-grained, and organic-rich sediment with an overlying water column of 2.5 m and two sites (Kairiai and Vente) had sandy sediments with an overlying water column of 1 m. At the deeper sites, muddy sediments were collected by using a hand-corer, whereas at sandy, shallower sites, they were collected by hand. Transparent Plexiglas liners having different dimensions (diameter × length) were used for benthic nutrient fluxes, aerobic and total respiration, and denitrification measurements (8 × 30 cm, *n* = 10 per sampling site) and for sediment characterization (5 × 30 cm, *n* = 5 per sampling site). Once collected, intact cores for flux measurements were leveled in order to have 10 cm of sediment and 15 cm of overlying water. All cores were submersed with the top open in boxes containing site water cooled with ice packs and brought to the laboratory within a few hours from sampling for further processing and incubation. In addition, ~50 L of water was collected from each site for core maintenance, preincubation, and incubation activities. During sampling, bottom water was characterized for temperature, salinity, dissolved O_2_, and irradiance (CTD 90, Sea & Sun Technology); in the laboratory, bottom water was analyzed for chlorophyll *a* (Lorenzen and Jeffrey, 1980) and dissolved inorganic nitrogen (DIN; see later for the methods).

### Sediment Features

The upper sediment layer (0–3 cm) was characterized for (i) bulk density, determined as the ratio between wet weight and volume (5 ml) of sediment; (ii) water content, determined after desiccation of a known fresh sediment volume at 60°C until constant weight; (iii) porosity, calculated as the ratio between the volume of water and that of fresh sediment; and (iv) OM, measured as percentage of weight loss on ignition (LOI; 450°C, 2 h) from dried sediment.

For the general composition of the benthic microalgal community, the 3-mm uppermost sediment layer was sliced and immediately frozen. The samples were thawed one by one and viewed by epifluorescence microscopy (Olympus BH-2, 250× and 500× magnification) to get an overall view of the composition and dominating algal groups; the cells were identified to the nearest possible taxon. No actual cell counts were made.

Pigments (chlorophyll *a* and phaeopigments) were extracted from about 1 g of sediment (*n* = 3) taken from the upper 1 cm with 5 ml of 90% acetone and determined fluorometrically according to Lorenzen and Jeffrey (1980). Total phytopigment concentrations were defined as the sum of chlorophyll *a* and phaeopigment concentrations (Pusceddu et al., 2009) and converted to carbon equivalents using a conversion factor of 40 μg of C μg^−1^ (de Jonge, 1980; Pusceddu et al., 2009).

Protein, carbohydrate, and lipid analyses of the sediment were carried out according to Marsh and Weinstein (1966); Gerchakov and Hatcher (1972); and Rice (1982), respectively. All biochemical analyses were carried out (*n* = 3) on the top 1 cm of the sediment. Protein, carbohydrate, and lipid concentrations were converted to carbon (C) equivalents by using the following conversion factors: 0.49, 0.40, and 0.75 mg C mg^−1^, respectively (Fabiano et al., 1995); and the sum of protein, carbohydrate, and lipid carbon was referred to as biopolymeric carbon (BPC; Pusceddu et al., 2009). The percentage contribution of total phytopigments to BPC is an estimate of the algal fraction of the organic material in the sediment (Pusceddu et al., 2009). In this study, we used the algal fraction of BPC and the protein to carbohydrate ratio as proxies of the whole nutritional quality of sediment OM (Pusceddu et al., 2009).

### Incubations for Gas and Nutrient Exchange

In the laboratory, intact cores were preincubated and incubated according to Dalsgaard et al. (2000). Batch incubations of sandy sediments in the laboratory do not allow to reproduce the effects of multidimensional advective transport of solutes, which may occur *in situ* and affect benthic processes. The Curonian Lagoon is in general a low-energy system, but advective transport in sandy sediments cannot be excluded. Briefly, all cores were transferred with the top open in four tanks containing aerated and well-mixed site water at *in situ* temperature. Half of the cores from each site were double-wrapped in aluminum foil in order to measure processes simultaneously in the light and in the dark. Each core was provided with a teflon-coated magnetic bar driven by an external motor at 40 rpm and suspended 10 cm above the sediment-water interface to avoid particle resuspension. Stirring units were on during preincubation and incubation periods. After overnight preincubation, the water in the tank was exchanged; then the cores were closed with gas tight lids and incubated in the light and in the dark for flux measurements of dissolved O_2_, total inorganic carbon (TCO_2_), and inorganic N forms (NH_4_^+^, NO_2_^−^, and NO_3_^−^). Irradiance at the sediment surface reflected that measured *in situ* and was obtained by halogen lamps and screens. In spring, reproduced irradiance was 150 and 90 μE m^−2^ s^−1^ and in summer 120 and 60 μE m^−2^ s^−1^ at shallower sandy and deeper muddy sites, respectively. In both periods, incubation time was set to keep O_2_ concentration at the end of the experiment within ±30% of the initial value. To this purpose, O_2_ was frequently monitored *via* microelectrodes (OXY50, Unisense, DK) inserted through the lids. Cores were incubated for 6 h in spring (water temperature 2.5°C) and for 2 h in summer (water temperature 19°C). Water samples (60 ml) were collected at the beginning and at the end of the incubations using plastic syringes from each core water phase (~750 ml).

Samples for O_2_ and TCO_2_ determinations were transferred to 12-ml exetainers (Labco Ltd). Winkler reagents were added immediately to the O_2_ samples, which were titrated within 2 days (Strickland and Parsons, 1972). Samples for TCO_2_ were immediately titrated with 0.1 N of HCl (Anderson et al., 1986). Samples for nutrients were filtered through Whatman GF/F glass fiber filters into 20-ml plastic vials and frozen (−80°C) until analysis. NH_4_^+^ was determined spectrophotometrically according to Bower and Holm-Hansen (1980). NO_3_^−^ was determined after reduction to NO_2_^−^ in the presence of cadmium, and NO_2_^−^ was determined spectrophotometrically using sulfanilamide and *N*-(1-naphtyl) ethylenediamine (Golterman et al., 1978).

Fluxes of dissolved gases and nutrients were calculated from the change in concentrations in the cores with time and expressed on areal basis (μmol m^−2^ h^−1^ or mmol m^−2^ h^−1^). Negative values indicate fluxes from the water column to the sediment, whereas positive values indicate fluxes from the sediment to the water column. Daily fluxes were obtained by multiplying hourly fluxes measured in the light and in the dark by the number of light and dark hours of the corresponding sampling period, assuming constant rates.

Theoretical nitrogen uptake (TNU) by benthic algae, including settled phytoplankton and microphytobenthos, was calculated from net O_2_ fluxes measured in the light, assuming a photosynthetic quotient of 1.2 and an average C:N molar ratio for benthic and pelagic microalgae of 8 ± 1 (Hillebrand and Sommer, 1997). TNU may underestimate real assimilation rates, as a fraction of the O_2_ produced by benthic algae is not evolved to the water column but to the sediments where it is consumed by heterotrophs or by chemical oxidations (Zilius et al., 2012).

### Denitrification Measurements

The same set of cores used for solute fluxes underwent a sequential incubation to measure denitrification (light and dark rates) *via* isotope pairing technique (IPT; Nielsen, 1992). The second incubation was carried out a few hours after the end of flux incubation. In between incubations, all cores were submersed open in fresh, *in situ* well-mixed, and aerated water, maintaining the stirring on to renew the core water phase. Sequential incubations minimize the effects of core aging and allow reliable comparison of aerobic and total respiration and inorganic N fluxes with rates of denitrification.

The IPT allows partitioning total denitrification (D_tot_) into denitrification of NO_3_^−^ diffusing to the anoxic sediment from the water column (D_w_) and denitrification of NO_3_^−^ produced within the sediment due to nitrification (D_n_). Methodological concerns have been raised about the IPT due to the co-existence of anaerobic ammonium oxidation (anammox), which cannot be discriminated from denitrification as a source of N_2_ and makes invalid the assumptions on which IPT calculations are based (Robertson et al., 2019). In order to validate the IPT assumptions, different amounts of ^15^NO_3_^−^ from a 15 mM Na^15^NO_3_ solution (98 atom %, Sigma-Aldrich, St. Louis, MO, USA) were added to the water column of each of the five replicate cores to perform a concentration series experiment (Dalsgaard et al., 2000). Final ^15^NO_3_^−^ concentrations were 10, 20, 50, 75, and 100 μM in the spring and 5, 10, 15, 20, and 30 μM in the summer, due to different bottom water ^14^NO_3_^−^ concentrations in the two seasons. Before and few minutes after the ^15^NO_3_^−^ addition, water samples (10 ml) were collected from each core to calculate the ^14^NO_3_^−^ to ^15^NO_3_^−^ ratio in the water column. Top lids were positioned after 30 min from the ^15^NO_3_^−^ addition in spring and after 5 min in summer due to different O_2_ penetration in sediments; incubation time was identical to that of flux measurements (6 and 2 h in spring and in summer, respectively). At the end of the incubation, 5 ml of ZnCl_2_ (7 M) was added to the water phase of each core, and then sediment and water were gently slurred. An aliquot of the slurry was transferred to a 12-ml exetainer; ^14^N^15^N and ^15^N^15^N abundance in N_2_ was analyzed by gas chromatography-isotopic ratio mass spectrometry (GC-IRMS) at the National Environmental Research Agency, Silkeborg, Denmark. As the genuine ^28^N_2_ production was independent from the concentration of the added ^15^NO_3_^−^, denitrification rates were calculated according to the equations and assumptions of Nielsen (1992). Denitrification efficiency, defined as the percentage of total processed inorganic N released as N_2_, was calculated as the ratio between N-N_2_ fluxes and the sum of N-N_2_ fluxes plus N-DIN effluxes from sediments.

Dark denitrification rates of water column NO_3_^−^ were also estimated from sediment O_2_ demand and bottom water concentrations of NO_3_^−^ and O_2_ with the equation proposed by Christensen et al. (1990):

$$D_{W}=F_{O_{2}}\cdot \alpha \cdot \left[\sqrt{\left(1+\frac{D_{{\mathrm{NO}}_{3}{}^{-}}}{D_{O_{2}}}\cdot \frac{C_{{\mathrm{NO}}_{3}{}^{-}}}{C_{O_{2}}}\cdot \frac{1}{\alpha }\right)}-1\right]$$

where $F_{O_{2}}$ is the dark sediment O_2_ demand, *α* is the ratio between depth-specific denitrification and O_2_ consumption activity (~0.8; Christensen et al., 1990), $D_{{\mathrm{NO}}_{3}^{-}}$ and $D_{O_{2}}$ are the diffusion coefficients of NO_3_^−^ and O_2_, and $C_{{\mathrm{NO}}_{3}^{-}}$ and $C_{O_{2}}$ are the water column NO_3_^−^and O_2_ concentrations, respectively.

### Statistical Analyses

Differences between fluxes of dissolved gas and nutrients, and rates of denitrification were analyzed by three-way analysis of variance (ANOVA), with time (T, *n* = 2; spring vs. summer), sediment type (S, *n* = 2; sand vs. mud), and incubation condition (IC, *n* = 2; light vs. dark) as factors. The factor S contrasted two groups, as data from the deeper muddy stations (Arkliu Ragas and Nida) and from the shallower sandy stations (Kairiai and Vente) were pooled. Differences between the denitrification to sediment O_2_ demand ratios were analyzed by two-way ANOVA with time (T, *n* = 2; spring vs. summer) and sediment type (S, *n* = 2; sand vs. mud) as factors.

Variations in biopolymers’ contents in the top 1 cm of the sediment were assessed using a three-way ANOVA, with time (T, *n* = 2; spring vs. summer) and sediment type (S, *n* = 2; sand vs. mud) as fixed crossed factors, and site (ST, *n* = 2) nested in S, with *n* = 3 as the combination of factors. Prior to the analyses, homogeneity of variance was tested using the Cochran test, and wherever needed, the data transformed to meet the ANOVA assumptions. When significant differences among levels of the fixed factors were observed, Student-Newman-Keuls (SNK) post-hoc comparison tests were also carried out. To test variations in the biochemical composition of sedimentary OM, a distance-based permutational multivariate ANOVA (PERMANOVA; Anderson, 2001; McArdle and Anderson, 2001) was applied. The analysis was based on Euclidean distances of previously normalized data, using 999 random permutations of the appropriate units (Anderson and ter Braak, 2003). The observed differences were finally visualized using bi-plots derived after a canonical analysis of principal coordinates (CAP), aimed at assigning times, sediment type, and sampling sites to categories identified *a priori*. Both PERMANOVA and CAP analysis were carried out, under the same design described above, by means of the PERMANOVA and CAP routines, respectively, included in the Primer 6+ package (Clarke and Gorley, 2006).

To assess whether and how much the composition and (nutritional) quality of sedimentary OM explained changes in the benthic fluxes and in the rates of denitrification, non-parametric multivariate multiple regression analyses that were based on Euclidean distances were carried out using the routine DISTLM forward (McArdle and Anderson, 2001). The forward selection of the predictor variables was carried out with tests by permutation. *p* values were obtained using 999 permutations of raw data for the marginal tests (tests of individual variables), while for all of the conditional tests, the routine uses 999 permutations of residuals under a reduced model. Predictor arrays of variables included the following: (i) protein, carbohydrate, lipid, and phytopigment sedimentary contents (composition); and (ii) protein to carbohydrate ratio values, and protein and algal fractions of biopolymeric C (nutritional quality). Statistical significance was set at *p* < 0.05.

## Results

### Water and Sediment Features

In spring, the mean chlorophyll *a* concentration in the lagoon water column was 23 ± 8 μg L^−1^ and the phytoplankton assemblages were dominated by diatoms (70–80%), whereas in summer, chlorophyll *a* concentration averaged 56 ± 11 μg L^−1^ with a dominance of cyanobacteria (60–85%; Table 1; Giardino et al., 2010). At the sampling sites, NO_3_^−^ concentrations were 30–60 times higher in spring (87–198 μM) than in summer (3–5 μM), whereas NH_4_^+^ concentrations remained similar in the two seasons (2–4 μM; Table 1).

**Table 1:** Physico-chemical characterization of bottom water at the four sampling sites.

Bottom water		March	July
Irradiance (μE m^−2^ s^−1^)	Arkliu R.	96	63
	Nida	82	55
	Kairiai	148	115
	Vente	154	126
Temperature (°C)	Arkliu R.	2.4	19.0
	Nida	2.6	19.2
	Kairiai	2.5	18.8
	Vente	2.4	18.9
Conductivity (μS cm^−1^)	Arkliu R.	343	312
	Nida	360	305
	Kairiai	310	290
	Vente	290	275
Dissolved O_2_ (μM)	Arkliu R.	380	269
	Nida	371	255
	Kairiai	410	271
	Vente	395	278
Chlorophyll *a* (μg L^−1^)	Arkliu R.	24.2 ± 3.4	61.7 ± 6.6
	Nida	22.5 ± 4.4	56.7 ± 4.6
	Kairiai	19.1 ± 2.6	49.3 ± 8.0
	Vente	21.9 ± 3.9	52.4 ± 3.9
NH_4_^+^ (μM)	Arkliu R.	4.5 ± 0.2	2.4 ± 0.2
	Nida	2.0 ± 0.1	3.9 ± 0.2
	Kairiai	1.9 ± 0.3	4.2 ± 0.3
	Vente	2.1 ± 0.4	2.2 ± 0.2
NO_3_^−^ (μM)	Arkliu R.	86.5 ± 1.3	3.1 ± 0.6
	Nida	198.0 ± 2.3	3.2 ± 0.2
	Kairiai	87.0 ± 0.9	4.5 ± 0.3
	Vente	126.0 ± 1.7	4.8 ± 0.4

**Figure 2:**
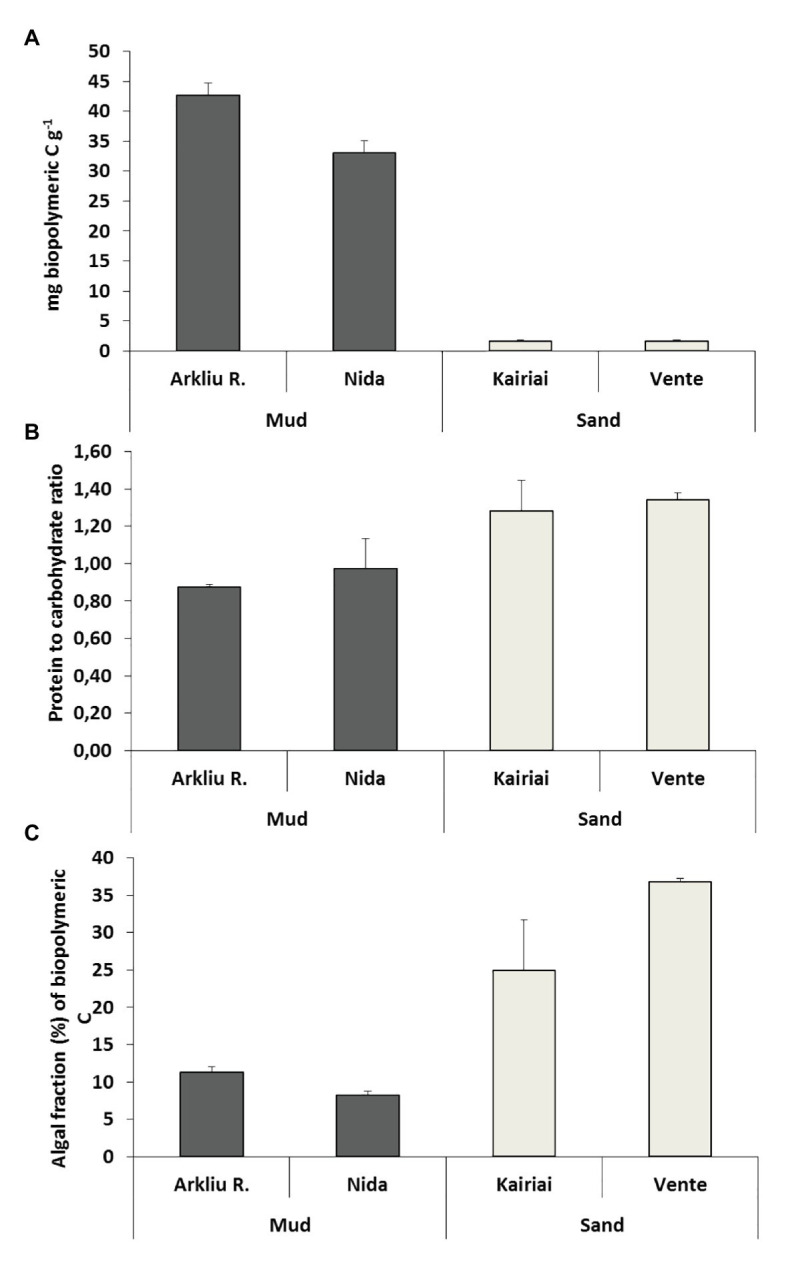
The three panels report the biopolymeric carbon content (A), the protein to carbohydrate ratio (B), and the algal fraction of biopolymeric carbon (C) in the sediments of the four sampling sites. Average values (±standard error, *n* = 3) are reported.

**Figure 3:**
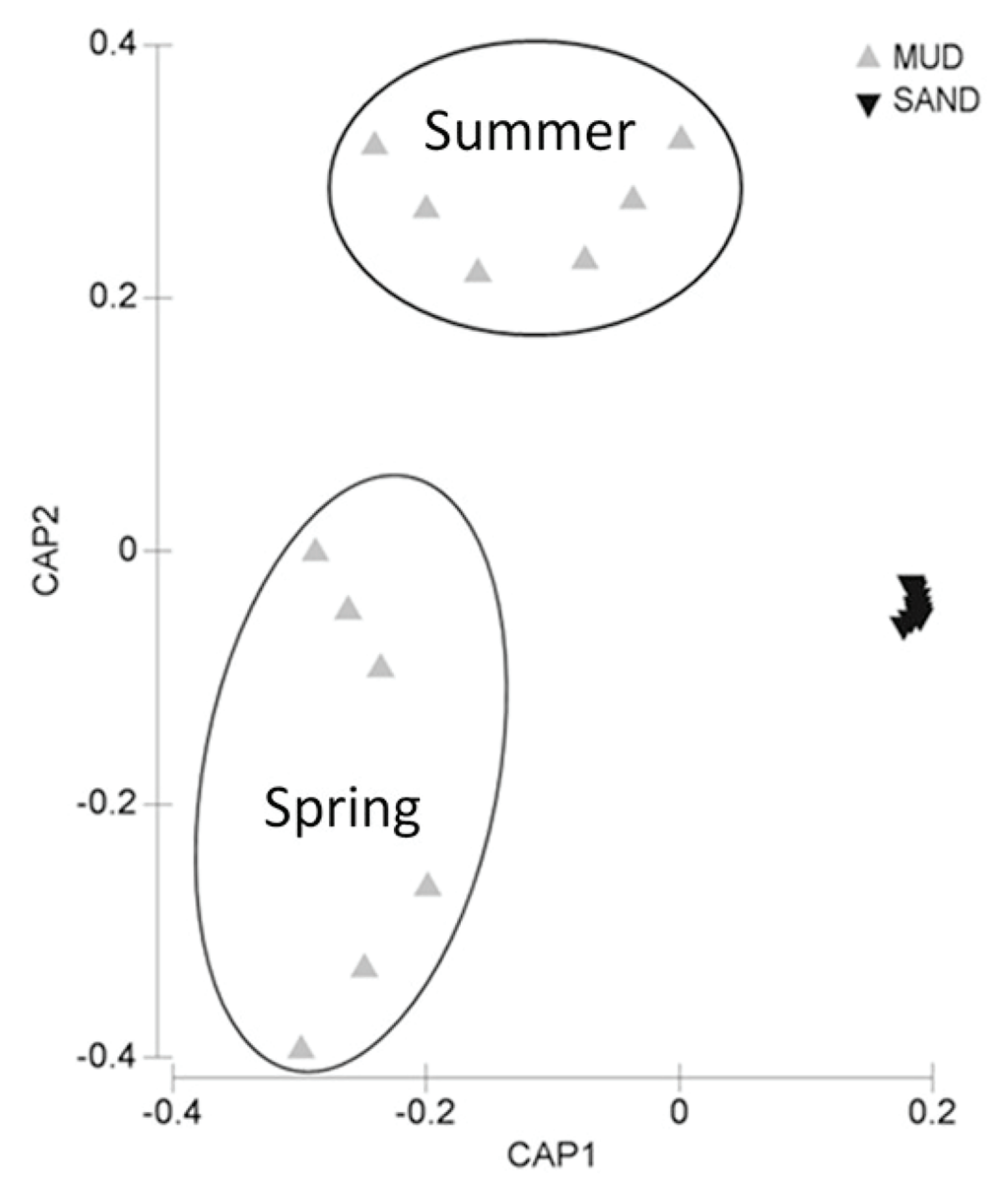
Bi-plot after the canonical analysis of principal coordinates (CAP) illustrating differences in the biochemical composition of sedimentary organic matter in the two sediment types.

Muddy sediments (Arkliu Ragas and Nida sites) had an OM content (LOI) of ~20%, whereas sandy sediments (Kairiai and Vente sites) had an OM content of ~1%. Similar differences were revealed by pigments and biochemical sediment analyses, with concentrations of chlorophyll *a* and BPC in muddy sediments (70–120 μg Chl *a* g^−1^ and 30–40 mg C g^−1^, respectively) exceeding those in sandy sediments by a factor of 5–30 (Figure 2A). The three-way ANOVA revealed that, except for lipids, sedimentary variables did not vary between sampling periods (ANOVA, *p* > 0.05) but varied among sites. Lipids displayed the highest concentrations at Arkliu Ragas (mud) where values had increased significantly from spring (11.9 ± 1.2 mg g^−1^) to summer (16.4 ± 1.9 mg g^−1^). Almost all sedimentary variables, except for the protein fraction of biopolymeric C (Figure 2B), showed significant differences between the two sediment types, with values in muddy sediments higher than those in sandy sediments (ANOVA and SNK tests, *p* < 0.05; Figure 2). The values of the protein to carbohydrate ratio and the algal fraction of biopolymeric C were significantly higher in sandy than in muddy sediments, consistently in both sampling periods (ANOVA and SNK tests, *p* < 0.01; Figures 2B,C). The PERMANOVA test revealed no significant temporal changes in the biochemical composition of sedimentary OM at sandy sites, whereas, as illustrated also by the CAP bi-plot (Figure 3), it revealed the presence of striking biochemical differences at muddy sediments in the two sampling periods.

The deeper muddy sites were characterized by the exclusive presence of settled, though vital, planktonic diatom species of the genera *Thalassiosira*, *Cyclotella*, *Coscinodiscus*, and *Fragilaria* or by green algae of the genera *Scenedesmus* and *Pediastrum*, whereas no benthic microalgal species were present. In contrast, at the two shallower sandy sites, a well-developed microphytobenthic flora was present in spring, when two *Navicula* species dominated at Kairiai, while at Vente, the community was more diverse. Some planktonic diatoms were also present at both sandy sites. In summer at Kairiai, epipelic diatoms together with the benthic cyanobacterium *Merismopedia* sp. co-dominated the sediment algal assemblages. At Vente, epipsammic diatoms were more abundant than at Kairiai.

### Oxygen and Total Inorganic Carbon Fluxes

**Figure 4:**
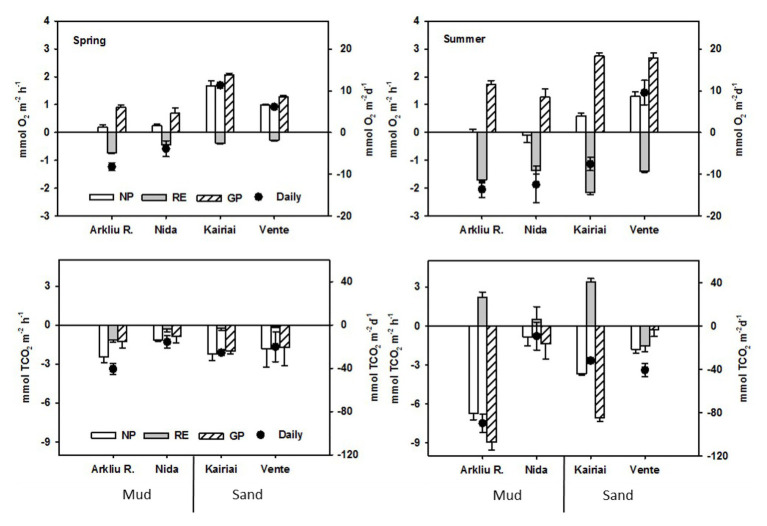
Dissolved O_2_ and inorganic carbon (TCO_2_) fluxes measured at the four study sites in spring and summer 2009 in the light (NP, net production) and in the dark (RE, respiration). Gross production (GP) was calculated as NP-RE; net daily budgets were calculated multiplying NP and RE by the corresponding number of light and dark hours in a day. Reported rates are averages ± standard errors (*n* = 5).

In spring, the daily O_2_ flux was negative at muddy sites (−8.20 ± 0.42 and −3.87 ± 0.34 mmol O_2_ m^−2^ day^−1^ at Arkliu Ragas and Nida, respectively), whereas sandy sites acted as O_2_ sources (11.36 ± 0.86 and 6.16 ± 0.29 mmol O_2_ m^−2^ day^−1^ at Kairiai and Vente, respectively; Figure 4). In summer, O_2_ uptake increased by a factor of 2.7 and 5.3 at the muddy and sandy sites, respectively. As in spring, net and gross primary production was higher at the two sandy sites, but only the Vente site was daily autotrophic. The three-way ANOVA showed significant interactions (*p* < 0.01) between the factors sediment type and incubation condition and between incubation condition and time; i.e., differences between sediment types and time depended on the light or dark regimes.

The fluxes of TCO_2_ were only partially coupled to those of O_2_ (Figure 4). In both sampling periods, TCO_2_ fluxes displayed a pronounced, light-dependent uptake, even if negative fluxes were measured also in the dark (Figure 4). In spring, the benthic photosynthetic quotient (O_2_ to TCO_2_ flux ratio in the light) averaged 0.18 (pooled data from all sites), suggesting that only a minor fraction of the O_2_ produced during photosynthesis was released to the water column or that other processes apart from photosynthetic C-fixation were responsible for a fraction of TCO_2_ uptake. In summer, the benthic respiratory quotient (TCO_2_ to O_2_ dark flux ratio, calculated pooling data from Arkliu Ragas, Kairiai and Nida sites) was higher than 1 and suggested an accumulation of anaerobic metabolism end-products. Daily fluxes of TCO_2_ were negative at all sampling sites and exhibited a similar pattern among stations in the two seasons; both the highest (Arkliu Ragas) and lowest (Nida) daily fluxes were found in muddy sediments (Figure 4). The three-way ANOVA indicated significant differences between incubation conditions, although differences depended on the sampling period (ANOVA, *p* < 0.01).

### Nutrient Fluxes

**Figure 5:**
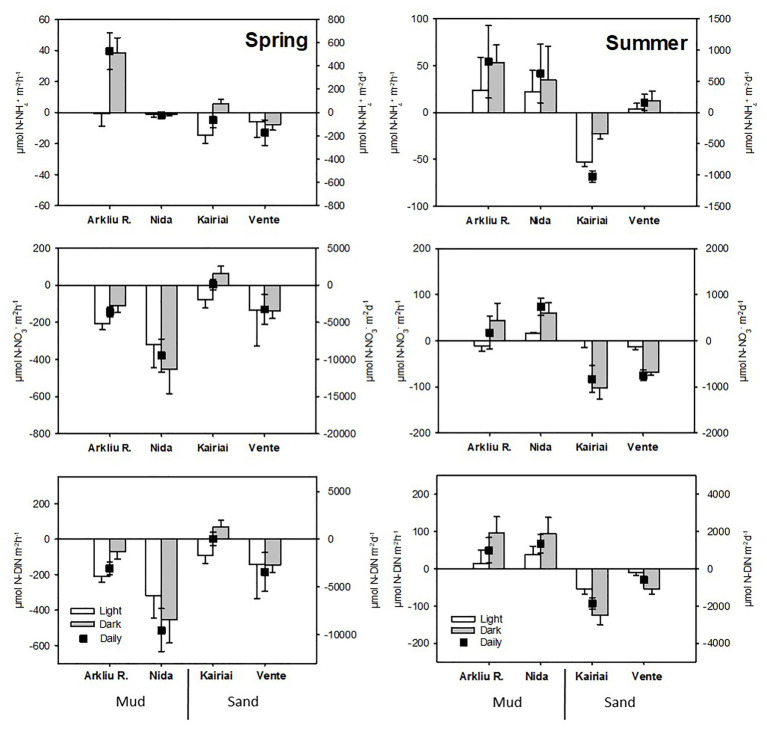
Light, dark, and daily flux across the sediment-water interface of NH_4_^+^, NO_3_^−^, and DIN (average ± standard error, *n* = 5) measured in spring and summer 2009 at the four sampling sites. Note different scales on the y-axes.

Some general patterns appeared for nutrient fluxes. On a daily basis, sandy sediments acted mostly as net sinks for DIN, while muddy sediments switched from DIN sinks (spring) to DIN sources (summer; Figure 5). Fluxes of NO_3_^−^ were larger in spring than in summer and mostly directed towards the sediment, whereas fluxes of NH_4_^+^ were higher in summer and mostly directed out of the sediment, except for the sandy Kairiai site (Figure 5). Fluxes of NO_2_^−^ were generally below 1 μmol NO_2_^−^ m^−2^ h^−1^ (data not shown). The effect of sediment type depended on the sampling period for both NH_4_^+^ and NO_3_^−^ fluxes (ANOVA, *p* < 0.05). In spring, muddy sediments displayed high uptake of NO_3_^−^ sustained by its large availability in the bottom water, whereas lower uptake or slight production was found at the two sandy sites (Kairiai and Vente). In summer, considerably decreased NO_3_^−^ concentration resulted in much lower rates of NO_3_^−^ consumption at all sites (Figure 5). There was always a significant effect of light on NH_4_^+^ fluxes, with lower efflux or higher uptake by the sediment in the light (ANOVA, *p* < 0.05). Overall, NO_3_^−^ fluxes drove most of DIN fluxes in both sampling periods and at all study sites.

### Denitrification Rates

**Figure 6:**
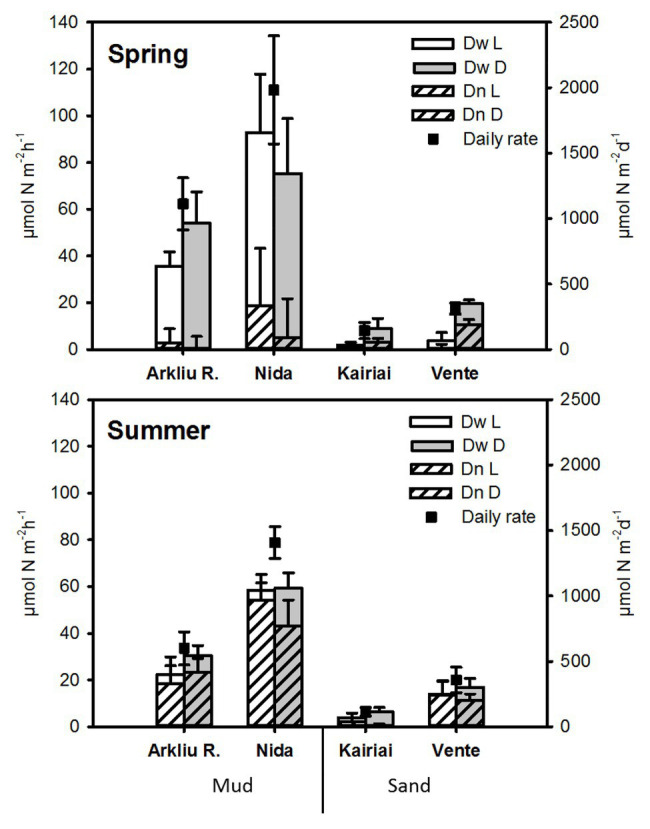
Rates of denitrification measured in spring and summer 2009 in the Curonian Lagoon sediments. Light and dark rates of denitrification of nitrate (NO_3_^−^) diffusing to anoxic sediments from the water column (D_w_) and of NO_3_^−^ produced *via* nitrification within sediments (D_n_), together with daily rates (average ± standard error, *n* = 5), are reported.

In all experiments, genuine ^28^N_2_ production was independent from ^15^NO_3_^−^ amendments, suggesting negligible contribution of anammox to N_2_ production. Total denitrification (D_tot_ = D_w_ + D_n_) varied between 0.6 and 135.5 μmol N m^−2^ h^−1^ in spring and between 2.4 and 67.5 μmol N m^−2^ h^−1^ in summer (Figure 6). Muddy sites exhibited higher D_tot_ rates than did sandy sites in both seasons. In spring, daily N_2_ production *via* D_tot_ varied between 1.1 and 2.0 mmol N m^−2^ day^−1^ at muddy sites, whereas it was about one order of magnitude lower, between 0.14 and 0.31 mmol N m^−2^ day^−1^, at sandy sites. In summer, D_tot_ rates measured at muddy sites decreased significantly as compared with the values measured during spring, whereas at sandy sites, D_tot_ remained similar in the two seasons. The relative contribution of D_w_ and D_n_ to D_tot_ were different between muddy and sandy sites, but differences depended upon the sampling period (ANOVA, *p* < 0.01). In spring, denitrification was sustained mainly by D_w_, representing 85–90% and 60–76% of D_tot_ in mud and sand, respectively. In summer, D_n_ dominated (>80% of D_tot_, with Kairiai as only exception). In both seasons, denitrification rates did not vary significantly between light and dark conditions (ANOVA, *p* > 0.05).

### Nitrogen-Related Benthic Processes in the Light

Theoretical inorganic N uptake (TNU), total denitrification rates, and net dissolved inorganic N fluxes measured in light incubations during the two sampling periods are reported in Table 2. In spring, at the deeper muddy sites Arkliu Ragas and Nida, TNU by benthic algae was much lower than measured fluxes of DIN, which were mostly sustained by NO_3_^−^ demand. Rates of N removal *via* denitrification were comparable (Arkliu Ragas) or higher (Nida) than TNU rates. In the same period, at shallow sandy sites, TNU rates were similar to (Vente) or exceeded (Kairiai) measured DIN fluxes. At sandy sites, the vast majority of inorganic N fluxes were channeled to the biomass of benthic algae *via* uptake, whereas the fraction that was denitrified was comparatively insignificant.

**Table 2:** Calculated theoretical inorganic nitrogen (N) uptake by benthic microalgae (TNU), measured N loss *via* total denitrification, and measured net fluxes of dissolved inorganic nitrogen (DIN) during light incubations in spring and summer 2009 at the four sampling stations.

		TNU (μmol N m^−2^ h^−1^)	Total denitrification (μmol N m^−2^ h^−1^)	Net DIN flux (μmol N m^−2^ h^−1^)
Spring	Arkliu R.	−24.7 ± 7.1	35.7 ± 2.8	−209.0 ± 24.8
	Nida	−31.9 ± 6.3	92.9 ± 14.7	−319.2 ± 77.5
	Kairiai	−223.3 ± 14.9	1.9 ± 0.9	−92.5 ± 33.2
	Vente	−133.2 ± 2.4	3.9 ± 2.1	−140.0 ± 120.8
Summer	Arkliu R.	−0.6 ± 10.8	22.3 ± 3.0	13.4 ± 27.5
	Nida	0	58.3 ± 2.4	37.8 ± 17.3
	Kairiai	−80.3 ± 25.4	4.0 ± 1.2	−53.2 ± 11.8
	Vente	−172.3 ± 17.9	14.0 ± 2.0	−9.4 ± 7.6

In summer at muddy sites, limited light penetration and benthic O_2_ production resulted in low TNU. The absence of a DIN sink at the sediment-water interface and the increase of mineralization rates reversed the flux of inorganic N, which became positive. At muddy sites, denitrification drove a major fraction of benthic N fluxes; however, it decreased compared with that in spring, likely due to decreased NO_3_^−^ concentration in the water column and limited nitrification. At sandy sites, in summer, TNU by microphytobenthos remained the most important inorganic N flux, and denitrification remained a scarcely relevant process. The net DIN flux to the sediments represented a fraction of theoretical N requirements by benthic algae, in particular at Vente (Table 2).

## Discussion

### Quantity, Biochemical Composition, and Nutritional Quality of Sediment Organic Matter and Its Relationships With Benthic Processes

Our study identified clear and statistically significant differences in the quantity and composition of sedimentary OM between muddy and sandy sediments. Compiled data on biopolymeric C concentration of sediments from different oceanic and coastal regions were significantly correlated with the total organic carbon content, the amount of phytopigments, and benthic O_2_ consumption (Pusceddu et al., 2009). Concentrations of BPC of >2.5 mg C g^−1^ were defined as a threshold level for eutrophic systems, in which heterotrophs may experience mostly refractory organic carbon (Pusceddu et al., 2009). This threshold sets a large difference between sandy (1.3–1.9 mg biopolymeric C g^−1^) and muddy stations (30–40 mg biopolymeric C g^−1^) in the Curonian Lagoon, with the latter stations hypertrophic and under the risk of hypoxic or anoxic events (Pusceddu et al., 2007). Such accumulation of OM is likely due to the confined location of stations Nida and Arkliu Ragas relative to the Nemunas River-driven lagoon currents, to the long residence time of water and the frequent algal blooms (Umgiesser et al., 2016). It may also depend upon uncoupled input and mineralization rates of the OM, due to absence of bioturbation, limited O_2_ penetration, and dominance of anaerobic microbial metabolism (Zilius et al., 2012).

Muddy sediments had significantly higher OM content, were more heterotrophic, and removed more N *via* denitrification than sandy sediments. Muddy sediments hosted vital algal cells, although settled from the water column, and reflected the composition and the OM nutritional quality of water column particles. They displayed seasonally significant biochemical differences, with an increase of lipids during summer. Such increase is likely the result of the seasonal shift from diatom to cyanobacteria-dominated phytoplankton community and of the effects of light and nutrient limitation on phytoplankton macromolecular composition. Different studies have demonstrated indeed an increase in lipid synthesis and algal content under nutrient limitation, affecting the phytoplankton nutritional value (Harrison et al., 1990; Zeng et al., 2010).

Sandy sites are located in the so-called transitional area of the Curonian Lagoon, where the influence of the Nemunas River and high flushing rates set the dominant granulometry and prevent OM accumulation (Umgiesser et al., 2016). Sandy sites, due to the shallower water column and higher light availability, were colonized by an active layer of benthic microalgae and were mostly net autotrophic. Here, microphytobenthos represented the main driver of inorganic N benthic fluxes and the main N temporary sink, whereas denitrification had a comparatively minor importance. Our results suggest that the two sediment types differ significantly also in terms of OM nutritional quality. Muddy sediments appear indeed characterized by higher concentrations of biopolymers, but a nutritional quality lower than sandy sediments. This result is in good agreement with previous studies conducted in several Mediterranean coastal lagoons, where typically higher concentrations of sedimentary OM are coupled with more refractory detritus (Pusceddu et al., 2003, 2007, 2009). These striking differences in the quantity and biochemical composition of sedimentary OM between the two sediment types emphasize the potential effects of these trophic attributes of sediments on benthic N transformations.

**Figure 7:**
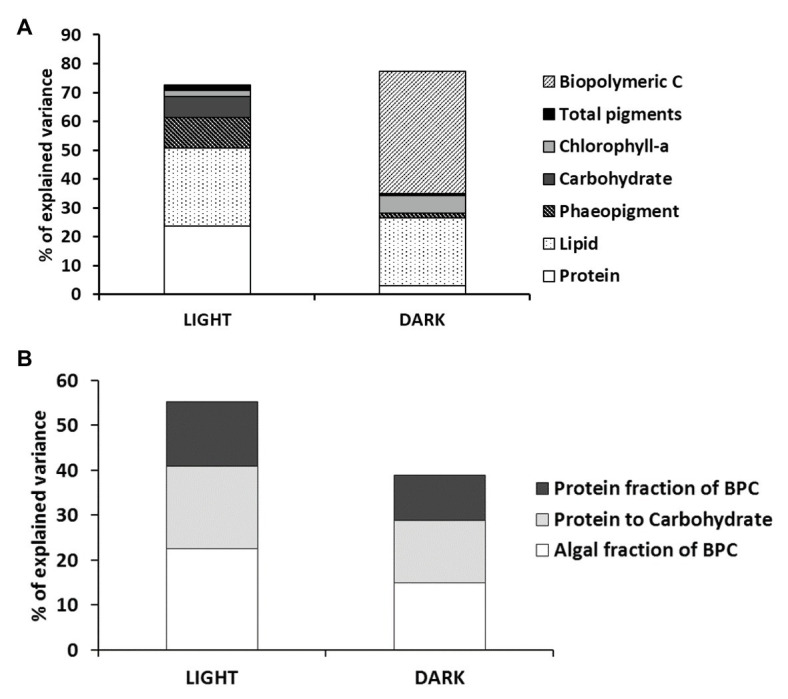
Results of the multivariate multiple regression analysis testing for the relationships between benthic processes and the quantity **(A)** and nutritional quality **(B)** of sedimentary organic matter. BPC, biopolymeric carbon.

The multivariate multiple regression analysis revealed that, overall, benthic processes under light and dark regimes were significantly related with the biochemical composition and nutritional quality of sedimentary OM. The quantity of OM explained about 70% of the total variance in benthic processes. Nevertheless, variables explaining significant proportions of benthic processes interestingly varied between the two light regimes (Figure 7A): in the light, protein and lipid concentrations explained together more than 50% of benthic processes variance, whereas in the dark, the biopolymeric C contents explained alone more than 40% of the total variance. In the light incubations, this can be explained by different protein and lipid syntheses in settled pelagic or by benthic diatoms under light or nutrient limitation conditions (Shifrin and Chisholm, 1981; Taguchi et al., 1987). In the dark, this can be explained in terms of total BPC availability for heterotrophs, affecting benthic respiration rates and nutrient recycling. The nutritional quality of sedimentary OM explained different proportions of variance in the benthic processes in the two incubation conditions (i.e., 55 and 39% in the light and in the dark, respectively), though the importance of the different descriptors of nutritional quality had a similar rank in explained variance (algal fraction of BPC > protein to carbohydrate ratio > protein fraction of BPC; Figure 7B). The relevance of the algal fraction of BPC as the factor explaining the variance observed in light measurements is obvious. The relevance of the other two factors probably reflects differences in the biochemical composition of microalgal communities at sandy and muddy sites recovered in the two sediment typologies (Harrison et al., 1990). The variance of processes measured in the dark is explained by the quality of sedimentary OM to a lesser extent than that in light incubations; such discrepancy is probably due to the role of other unaccounted factors like the limited O_2_ penetration or the toxicity of anaerobic metabolism end-products or the occasional presence of bioturbating macro or meiofauna, which might be more important under dark conditions (Zilius et al., 2012; Benelli et al., 2018).

### Benthic Nitrogen Uptake, Denitrification, and N_2_ Fluxes in Autotrophic and Heterotrophic Sediments

Shallow sandy sites were always releasing O_2_ to the water column during light incubations and were net autotrophic in spring (both sites) and in summer (only Vente). They were mostly sinks for inorganic N and displayed lower rates of denitrification than muddy sediments. This is in agreement with previous studies by Sundbäck et al. (2000); Bartoli et al. (2003); Risgaard-Petersen (2003); and Engelsen et al. (2008) for autotrophic sediments dominated by benthic microalgae. Net O_2_ production was also measured at muddy sites in spring, but the rates were low. This fact was probably due to higher depth and reduced light availability, elevated OM content, and elevated microbial respiration, determining the net heterotrophy of these enriched sediments in both sampling periods. The analysis of the microalgal assemblages revealed that muddy sites hosted only settled pelagic algae, probably because the reduced light availability strongly limited the development of microphytobenthos. As the average depth of the Curonian Lagoon is 3.8 m, shallow areas represent a minor fraction of the whole lagoon surface (<5%), and the water column chlorophyll *a* concentrations were exceptionally low in summer 2009 (Bresciani et al., 2012), we speculate that most of the Curonian Lagoon sediments are not illuminated and therefore heterotrophic (Zilius et al., 2014).

The spring sampling was characterized by elevated NO_3_^−^ availability in the water of all stations. At the deeper muddy sites Arkliu Ragas and Nida, the low benthic photosynthetic activity combined with the large excess of N resulted in a scarce competition between benthic primary producers and heterotrophs. At these sites, large DIN uptake by sediments was measured in light incubations, exceeding by a factor of ~l0 calculated N requirements to support benthic primary production and by a factor of 3–6 measured rates of denitrification. Nitrate ammonification, not measured in this study, or incorporation of N into microbial biomass may explain NO_3_^−^ consumption in excess to denitrification and uptake. In muddy sediments, these processes can be a consequence of the large pool of refractory OM (Nizzoli et al., 2010). High NO_3_^−^ and OM availability supports the elevated rates of N removal *via* denitrification measured in spring at 2.5°C, which is comparable (Arkliu Ragas) or higher (Nida) than N requirements by benthic primary producers. In the same season, at shallow sandy sites, high rates of primary production resulted in TNU rates similar (Vente) to or exceeding (Kairiai) measured DIN uptake. These results suggest that N requirements by benthic microalgae at sandy sites were sustained by N availability in both the water column and sediment pore waters. Despite high NO_3_^−^ concentrations in water, denitrification rates measured in sandy sites were insignificant compared with measured DIN uptake and TNU, likely due to much lower OM pool in sediments and inhibition of denitrification activity by microphytobenthos. Sandy sites had >10-folds lower concentration of OM than muddy sites, with a major proportion of the total biopolymeric C represented by the algal fraction (32–36% in sandy vs. 8–12% in muddy sediments).

In summer, denitrification rates measured in muddy sediments decreased as compared with spring rates, due to a combination of lower NO_3_^−^ availability in the water column, limited O2 penetration affecting nitrification, and different biochemical qualities of the sedimentary OM (Zilius et al., 2012). Benthic primary production and TNU rates decreased much more than denitrification during the warm season, and the latter process remained the most important benthic N flux. Increased NH_4_^+^ production within muddy sediments exceeded TNU and the weak N sink capacity of benthic algae. This resulted in net DIN regeneration, which decreased denitrification efficiency (24 and 38% at Arkliu Ragas and Nida, respectively, vs. 100% calculated for both sites in spring). In summer at sandy sites, total denitrification rates remained low but did not change significantly as compared with those in spring; what changed was the relative importance of D_w_ (higher in spring) and D_n_ (higher in summer) to the total process. Similar total denitrification in the two seasons was likely due to environmental changes resulting in contrasting effects on dissimilative N reduction as increased water temperature, increased ammonification, decreased water column NO_3_^−^, and decreased O_2_ penetration (Magri et al., 2020). At sandy sites, there were no seasonal differences in terms of quantity and macromolecular quality of the OM sedimentary pool, so this factor was likely not affecting denitrification. TNU by microphytobenthos was much larger than denitrification rates and measured DIN fluxes and remained the main sink of inorganic N. At Kairiai and Vente, the discrepancy between theoretical assimilation rates and net DIN fluxes suggests that pore water NH_4_^+^ or N-fixation represented an important source of N for the benthic microalgal communities (Zilius et al., 2020).

### The Regulation of Denitrification in the Curonian Lagoon

The regulation of denitrification in temperate estuarine systems with seasonally variable concentrations of NO_3_^−^ and variable sedimentary OM content and quality is a complex issue. In the Curonian Lagoon, NO_3_^−^ concentrations peaked during spring and D_w_ supported a major fraction of total denitrification in a period where, due to low water temperatures, and sediment O_2_ demand is low. Among others, Seitzinger (1994) and Racchetti et al. (2011) demonstrated that in wetland sediments, denitrification rates increased along with experimentally increased NO_3_^−^ concentrations in the overlying water, suggesting that NO_3_^−^ is an important factor controlling denitrification. However, in the Curonian Lagoon, water column NO_3_^−^ uptake is a partial predictor of total denitrification, as the latter can be sustained by nitrification in the sediments. During summer, temperature and sedimentary O_2_ consumption increased, but NO_3_^−^ concentrations were low to undetectable in the water column, and nitrification became a limiting step to supply NO_3_− to denitrifiers. As a result, summer rates of denitrification were similar to those measured in spring (sandy sediments) or lower (muddy sediments), despite significant differences of water temperature in the two seasons (~2.5 and ~19°C) and a 2–5 fold increase of sediment O_2_ demand. In moderately enriched sediments with low NO_3_^−^ concentration in the overlying water, denitrification is demonstrated to increase with OM, but it is better correlated with sediment O_2_ demand and therefore with the mineralization capacity of sediments and with nitrification rates (Seitzinger, 1994).

In the present study, dark denitrification to sediment O_2_ consumption ratios were significantly different among sediment types and seasons (two-way ANOVA, *p* < 0.01). In particular, ratios were always higher at muddy (0.123 ± 0.020 and 0.031 ± 0.005 in spring and summer, respectively) than at sandy sediments (0.056 ± 0.020 and 0.008 ± 0.001 in spring and summer, respectively) and in spring than in summer. This means that denitrification represented the major fraction of O_2_ uptake during winter at muddy sediments, whereas it represented the lowest fraction of O_2_ uptake during summer and at sandy sediments. These results support the complex regulation of denitrification in the Curonian Lagoon sediments, where NO_3_^−^ concentrations, sediment O_2_ demand, the OM content and quantity and the activity of benthic algae interact as factors. Eyre et al. (2013) report variable slopes (0.011–0.031) of regressions between dark N_2_ production (μmol N_2_-N m^−2^ h^−1^) and sediment O_2_ demand (μmol O_2_ m^−2^ h^−1^) in 12 benthic habitats within three different estuaries. Such variable slopes were correlated with OM C:N ratio and its *δ*^13^C signature, suggesting for the first time the importance of the origin and of the nutritional quality of the OM pool as regulators of denitrification.

**Figure 8:**
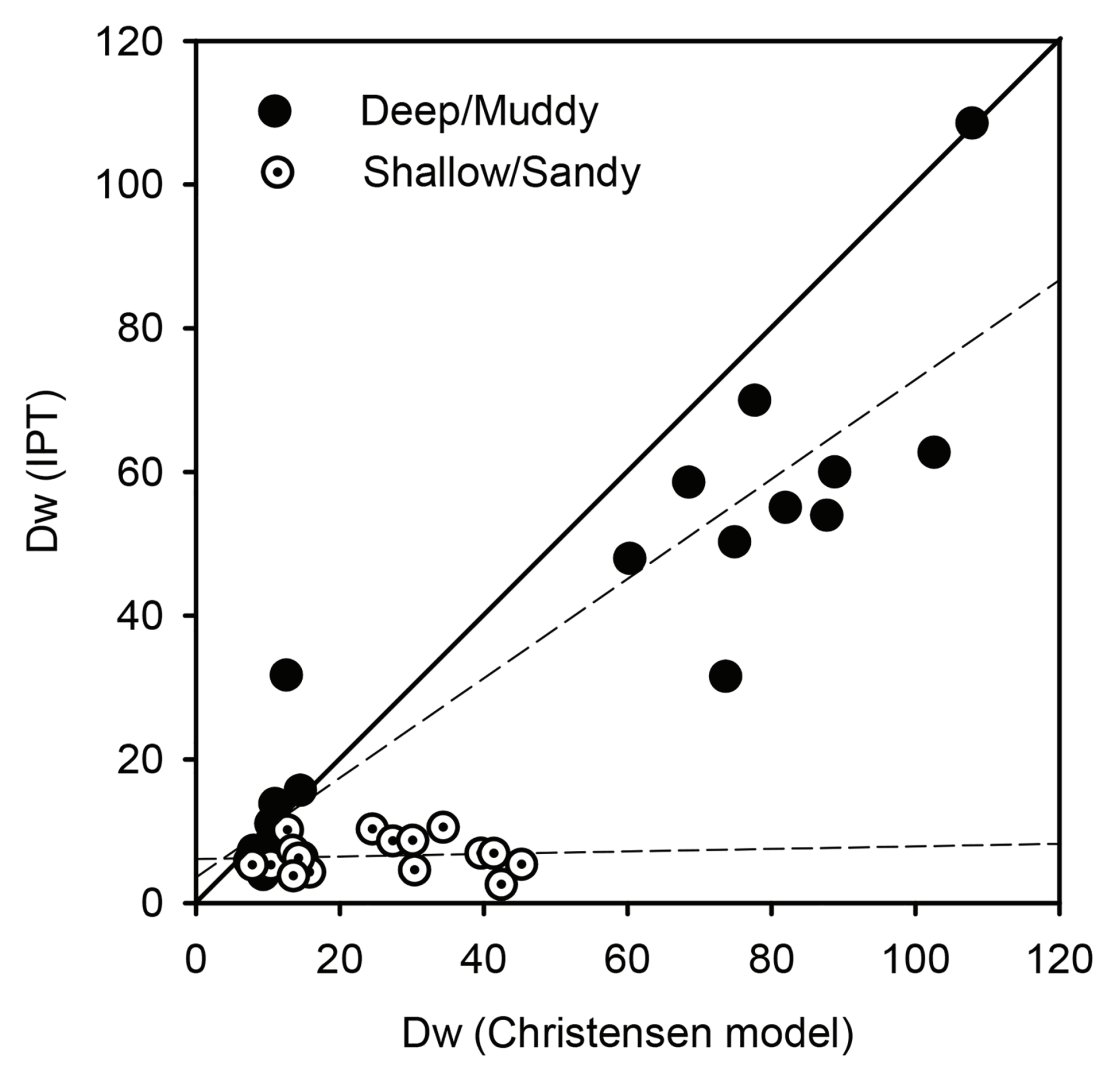
Predicted and measured rates of denitrification fuelled by NO_3_^−^ from overlaying water (D_w_) at deep/muddy and shallow/sandy sites in the Curonian Lagoon (pooled data from dark incubations of spring and summer 2009).

The equation proposed by Christensen et al. (1990), which calculates dark rates of denitrification of water column NO_3_^−^ from sediment O_2_ demand and NO_3_^−^ to O_2_ availability in bottom water, allows to compare measured and predicted D_w_ in the two sediment types. At muddy sites Arkliu Ragas and Nida, there was a good agreement between measured and predicted rates in both sampling periods (*R* ^2^ = 0.85, *p* < 0.001), whereas at sandy sites Kairiai and Vente, the regression between theoretical and measured rates was not significant (*R* ^2^ = 0.1, *p* = 0.67; Figure 8). In the latter sediment type, expected N removal in the dark, when microphytobenthos is not active, was much higher than measured rates, and this occurred in spring, with high NO_3_^−^ concentrations in the water, and in summer, when NO_3_^−^ concentrations dropped. This outcome supports the general inhibition of denitrification by microphytobenthos that was described by Henriksen and Kemp (1988) and Risgaard-Petersen (2003) in autotrophic estuarine sediments. Such inhibition seems independent from the illumination condition and from the inorganic N availability, as under inorganic N limiting conditions, dissolved organic N may represent an alternative important N source for benthic microalgae in sandy sediments, accounting for 50% of their N-demand (Sundbäck et al., 2011). Inhibition of denitrification in sandy sediment during spring and in the light period can be exacerbated by microphytobenthos activity, increasing O_2_ penetration in sediments and resulting in longer path for NO_3_^−^ to reach the denitrification zone (Sundbäck et al., 2000). During summer, when denitrification is mainly supported by D_n_, microphytobenthos activity can increase O_2_ penetration, but it also exerts a strong control of NH_4_^+^ supply to nitrifiers, as discussed by Risgaard-Petersen (2003). Such inhibition of denitrification by primary producers did not occur in muddy sediments hosting viable settled microalgae.

### Relevance of Sedimentary Denitrification in the Curonian Lagoon

Measurements of denitrification rates in boreal-temperate lagoons of the Baltic Sea are relatively scant, and the available dataset is limited (Silvennoinen et al., 2007). Spring denitrification rates measured in the Curonian Lagoon were typically two to three times higher than those found in the Baltic Sea Proper (<0.02–0.69 mmol N m^−2^ day^−1^, Tuominen et al., 1998), the Gulf of Finland (0.09–0.40 mmol N m^−2^ day^−1^, Hietanen and Kuparinen, 2008), the Archipelago Sea (0.09–0.91 mmol N m^−2^ day^−1^, Silvennoinen et al., 2007), the Himmerfjärden (0.1–0.42 mmol N m^−2^ day^−1^, Bonaglia et al., 2014), the Öre estuary (~0.14 mmol N m^−2^ day^−1^, Hellemann et al., 2017), and the Bothnian Bay (0.33–0.91 mmol N m^−2^ day^−1^, Silvennoinen et al., 2007). In the Curonian Lagoon, large spring rates of N removal were sustained by elevated riverine NO_3_^−^ concentrations (100 < [NO_3_^−^] < 200 μM) and loads (up to 100 tons N day^−1^; Vybernaite-Lubiene et al., 2017, 2018). High denitrification rates, similar to those reported in this study and among the highest reported for the Baltic area, were assumed for the Szczecin lagoon, which has also elevated external N loadings (0.98 and 3.72 mmol N m^−2^ day^−1^, Pastuszak et al., 2005). However, these rates need to be verified experimentally, as mass balances calculations can be affected by large uncertainties.

Recent studies demonstrated that anammox is a marginal process in the coastal area of Baltic Sea, that denitrification is mostly sustained by nitrification (D_n_) as D_w_ is limited by low NO_3_^−^ in the bottom water, and that denitrification is correlated with the organic carbon content of sediments (Jäntti et al., 2011; Bonaglia et al., 2014; Hellemann et al., 2017). The present study suggests that in the Curonian Lagoon, large seasonal and spatial variations in water-column NO_3_^−^ concentration and in sediment OM content and quality and the activity of microphytobenthos co-regulate the relative importance of D_w_ and D_n_ and determine different rates of total denitrification in sandy and muddy sites. OM content and NO_3_^−^ availability are recognized as major regulators of denitrification, whereas to our knowledge, OM quality as a regulator of benthic N cycling remains poorly explored (Christensen et al., 1990; Eyre and Ferguson, 2002; Pinã-Ochoa and Álvarez-Cobelas, 2006; Eyre et al., 2013).

In the Curonian Lagoon, benthic N-cycling underwent a strong seasonality. Denitrification (at muddy sediments) and benthic N uptake (at sandy sediments) were dominating processes in the spring, with high discharge and dominance by diatoms. During summer, with low discharge and dominance by cyanobacteria, microphytobenthos uptake continued to drive inorganic N fluxes at sandy sites, whereas at muddy sites, denitrification decreased and inorganic N was recycled to the water column. In spring, the average daily load of total N discharged in the Curonian Lagoon from the Nemunas River was estimated to be 97.3 ± 35.8 tons N day^−1^. Such amount averages recently measured riverine loads in March (2012–2016), which overlap those measured in the last 30 years (Vybernaite-Lubiene et al., 2018). As shallow areas cover a minor part (5%) of the Curonian Lagoon surface, denitrification rates measured at muddy sites were pooled, averaged, and upscaled. In spring, denitrification was estimated to remove 34.3 ± 13.0 tons N day^−1^ from the Curonian Lagoon. As a consequence, spring N loss *via* denitrification may represent up to 35% of the total N delivered from the Nemunas River. In the summer, a low-discharge period, daily average riverine N input to the lagoon was estimated to be 21.5 ± 4.2 tons N day^−1^ (averaged loads for July, Vybernaite-Lubiene et al., 2018) and decreased by ~80% as compared with the spring, a high-discharge period. Calculated N loss *via* denitrification in the summer (22.3 ± 6.7 tons N day^−1^) had also decreased, but only by 35% and was comparable with the N input from the Nemunas River. To our knowledge, only two annual N budgets (2012–2013) have been made for the Curonian Lagoon. They were made by comparing the loads from the Nemunas River with the loads from the lagoon to the Baltic Sea (Vybernaite-Lubiene et al., 2017). Such budgets suggested significant but variable reduction of NO_3_^−^ riverine loads within the lagoon (−59 and −31% in 2012 and 2013, respectively) and of total N in the 2 years (−14 and −19%, respectively). Vybernaite-Lubiene et al. (2017) showed that the largest decrease of NO_3_^−^ loads from the Nemunas River occurred in spring, which is in agreement with the high rates of denitrification of water column NO_3_^−^ that are reported in this study. During summer, riverine NO_3_^−^ concentrations were much lower, and denitrification was mostly sustained by nitrification.

The present work and that of Vybernaite-Lubiene et al. (2017) did not consider other potentially relevant N input terms to the lagoon, including diffuse sources, sewage treatment plants, the atmosphere, the adjacent Baltic Sea, and the fixation of N in the lagoon water column and in the sediments (Zilius et al., 2018). Extended reed belts and vegetated riparian areas along most of the lagoon perimeter should limit diffuse inputs to the Curonian Lagoon. Also, inputs from the Baltic Sea can be considered of minor importance, as marine inputs are occasional and limited to the lagoon northern area. The population living in Klaipeda (200,000 IE) generates at most 2.4 tons N day^−1^, assuming a standard pro capita N production of 12 g N IE^−1^ day^−1^. These N loads represent nearly 2 and 10% of riverine N inputs in spring and summer, respectively; but they are overestimated as the city of Klaipeda has a wastewater treatment plant operating denitrification. A similar amount of N is likely produced during summer by the fluctuating population of tourists visiting the Curonian Spit (150,000–250,000 units). If N loads from civil sources represent minor N inputs, the rates of summer fixation of N_2_ recently estimated by Zilius et al. (2020) in 42.4 ± 6.4 tons N day^−1^ represent an important N input term to the Curonian Lagoon. Such net import of N *via* fixation nearly doubles summer riverine loads and offset rates of N abatement *via* denitrification.

## Conclusion

Results from this study suggest pronounced seasonal differences between sandy autotrophic and muddy heterotrophic sites in the (i) nutritional quality and total amount of the OM in surface sediments; (ii) inorganic N uptake by benthic primary producers; and (iii) denitrification rates. In spring, sediments acted as inorganic N net sinks and N_2_ sources, due to the uptake by benthic algae (in particular at sandy sites) and denitrification of water column NO_3_^−^ (in particular at muddy sites). Shallow sandy sites had mats of active benthic algae that depressed denitrification rates due to strong control of nitrification or production of inhibitory compounds. This was not evident at deeper sites, which had only settled pelagic algae and were significant N_2_ sources. In summer, sandy sites continued to be temporary N sinks *via* microphytobenthos uptake, whereas at muddy sites, the drastic reduction of inorganic N availability in the water column resulted in lower rates of denitrification.

The analyses of the macromolecular composition of the OM revealed striking differences between sandy and muddy sites in both sampling periods, which are in agreement with differences in metabolic measurements. At sandy sites, the OM content was lower but had better quality than muddy sediments. Here, the bulk of OM mostly consisted of benthic microalgae, and the organic pool was similar in terms of macromolecular composition in both sampling periods. On the contrary, muddy sediments had much higher concentrations of OM, but with lower nutritional value.

The Nemunas River represents the largest freshwater and nutrient inputs to the lagoon and acts as an upstream regulator of the Curonian Lagoon benthic metabolism. Seasonally variable riverine N loads affect the competition between benthic microalgae and microbes and the rates of denitrification. Pronounced summer N-limitation and turbidity favor the shift from the dominance of diatoms to the dominance of cyanobacteria and affect the nutritional value of phytoplankton and the macromolecular composition of the sedimentary OM. The marked increase of lipids in muddy sediments probably reflects this seasonal shift, whose effects on benthic N cycling and specifically on denitrification need further investigations.

## Data Availability Statement

The original contributions presented in the study are included in the article/supplementary material, further inquiries can be directed to the corresponding author.

## Author Contributions

MBa, DN, AP, AR-B, and PV planned the activities. MBa, DN, AP, SB, MBr, and MZ performed the measurements. MBa, DN, AP, and SB performed the analysis and processed the experimental data. MBa, MZ, AP, SB, AR-B, and KS drafted the manuscript. All authors discussed the results and commented on the manuscript. All authors contributed to the article and approved the submitted version.

### Conflict of Interest

The authors declare that the research was conducted in the absence of any commercial or financial relationships that could be construed as a potential conflict of interest.
